# Assessment of Outliers and Detection of Artifactual Network Segments Using Univariate and Multivariate Dispersion Entropy on Physiological Signals

**DOI:** 10.3390/e23020244

**Published:** 2021-02-20

**Authors:** Evangelos Kafantaris, Ian Piper, Tsz-Yan Milly Lo, Javier Escudero

**Affiliations:** 1 School of Engineering, Institute for Digital Communications, University of Edinburgh, Edinburgh EH9 3FB, UK; javier.escudero@ed.ac.uk; 2 Usher Institute, Edinburgh Medical School, University of Edinburgh, Edinburgh EH16 4UX, UK; ian.piper@brainit.org (I.P.); mils.lo@ed.ac.uk (T.-Y.M.L.); 3 Royal Hospital for Sick Children, NHS Lothian, Edinburgh EH9 1LF, UK

**Keywords:** network physiology, dispersion entropy, multivariate analysis, outlier samples, data quality

## Abstract

Network physiology has emerged as a promising paradigm for the extraction of clinically relevant information from physiological signals by moving from univariate to multivariate analysis, allowing for the inspection of interdependencies between organ systems. However, for its successful implementation, the disruptive effects of artifactual outliers, which are a common occurrence in physiological recordings, have to be studied, quantified, and addressed. Within the scope of this study, we utilize Dispersion Entropy (DisEn) to initially quantify the capacity of outlier samples to disrupt the values of univariate and multivariate features extracted with DisEn from physiological network segments consisting of synchronised, electroencephalogram, nasal respiratory, blood pressure, and electrocardiogram signals. The DisEn algorithm is selected due to its efficient computation and good performance in the detection of changes in signals for both univariate and multivariate time-series. The extracted features are then utilised for the training and testing of a logistic regression classifier in univariate and multivariate configurations in an effort to partially automate the detection of artifactual network segments. Our results indicate that outlier samples cause significant disruption in the values of extracted features with multivariate features displaying a certain level of robustness based on the number of signals formulating the network segments from which they are extracted. Furthermore, the deployed classifiers achieve noteworthy performance, where the percentage of correct network segment classification surpasses 95% in a number of experimental setups, with the effectiveness of each configuration being affected by the signal in which outliers are located. Finally, due to the increase in the number of features extracted within the framework of network physiology and the observed impact of artifactual samples in the accuracy of their values, the implementation of algorithmic steps capable of effective feature selection is highlighted as an important area for future research.

## 1. Introduction

Network physiology, as a paradigm, aims to describe the interaction across diverse organ systems in the form of physiological networks. Within this framework, each system is represented as a node of the network, and interactions across systems are projected as edges between the nodes [1,2]. This approach allows the monitoring of complex physiological interactions in the body, through the detection of topological transitions which occur within the networks when their physiological state changes. Consequently, a vital step in this process is the utilization of methods that would allow the extraction of features for the characterization and monitoring of the investigated network. In its initial implementation, network physiology was used for the study of sleep stages and was limited in the utilisation of features extracted by the bivariate measurement of pair-wise coupling using the Time Delay Stability (TDS) method. TDS measures the time delay with which modulation in the output dynamics of a given node are consistently followed by corresponding modulations in the output of another node after appropriate preprocessing of the analyzed physiological signals [3]. The initial implementation of this framework was further expanded by recent research through the combination of network physiology and entropy quantification algorithms. Within this scope, non-linear entropy quantification algorithms have been used extensively due to their capacity for effective measurement of irregularity in biological signals [4,5]. Both univariate and multivariate algorithms have been applied to multivariate signals with limited preprocessing for the extraction of features from each node and from connections between two or more nodes, respectively [6,7].

Entropy quantification algorithms are based on Shannon entropy, the initial extension of the concept of Entropy to information theory by Shannon [8], and on Conditional Entropy, defined as the amount of information observed in a sample at a time point *n*, that cannot be explained based on previous samples from up to time point n−1 [9]. Univariate and multivariate algorithms based on Shannon Entropy, such as Dispersion Entropy (DisEn) [10,11,12] and Permutation Entropy (PEn) [13,14] alongside algorithms based on Conditional Entropy, such as Approximate Entropy (ApEn) [15], Sample Entropy (SampEn) [16,17], and Fuzzy Entropy (FuzzyEn) [18,19] have been implemented for the extraction of features from physiological recordings, to be used as nonlinear indexes for disease diagnosis and prognosis. However, while their utilisation within a network physiology paradigm could aid in the monitoring of physiological systems, the challenges introduced by the existence of artifactual outlier samples, which are a common occurrence in physiological recordings due to electromagnetic interference, loose equipment attachment, and user movement, have to be addressed [20,21,22]. When tested, the performance of the univariate ApEn, SampEn, and DisEn algorithms was significantly disrupted during the analysis of signal segments containing outlier samples [23,24,25].

For the purposes of this study, DisEn was selected for the extraction of univariate and multivariate features from formulated network segments. DisEn was selected due to its favorable performance characteristics, such as increased discrimination capacity and low computation time [11,12]. Since its original introduction in 2016 [10], novel variations have been proposed, such as Fluctuation-based Dispersion Entropy (FDisEn) [11], Reverse Dispersion Entropy (RDE) [26], and Reverse Fluctuation-based Dispersion Entropy (FRDE) [27]. In this study, the original DisEn is utilised due to the availability of an efficient multivariate implementation [12] and to test whether its sensitivity to artifactual outliers can be used as an advantage for the separation between high-quality versus artifactual network segments.

Addressing low data quality, arising from artifactual outliers, is a necessary step in order to ensure that algorithmic implementations based on network physiology are efficient at providing useful information when deployed in physiological monitoring applications. From an effectiveness standpoint, the performance of disease prognosis and clinical support algorithms can be severely limited when trained on noisy datasets [28,29]. Most importantly however, inaccuracy in the extracted features can lead to the already life-threatening phenomenon of “alarm fatigue” [30,31]. Alert systems currently deployed in intensive care units (ICU) are susceptible to producing excessive amounts of false positive alarms by mistaking artifactual outliers as indications that the patients are in a non-stable physiological state. The phenomenon of “alarm fatigue” is observed when clinical staff start ignoring alarms which they perceive as false, even when they are accurate, and as a result, put patients at risk [32,33]. While within the scope of network physiology, the characterization of the physiological state of individuals is not based simply on the univariate analysis of each separate physiological signal, but on the multivariate analysis of its representative network, it is important to ensure that deployed algorithms are capable of separating between viable network segments and network segments whose information content is disrupted from artifactual outliers. This is a prerequisite step prior to extracting insights concerning the physiological state of monitored individuals.

With this study, we aim to address the challenge of artifactual outliers in the utilisation of entropy quantification algorithms within a network physiology framework. The main objectives of the presented work are:

- The quantification of the effect of artifactual outliers in the accuracy of univariate and multivariate DisEn feature values extracted from physiological network segments.
- The assessment of whether a simple logistic regression classifier could be effectively trained on distributions of features extracted from “normal” and “artifactual” network segments to differentiate between the two.

For these objectives, network segments are formulated from four synchronised physiological time-series: electroencephalogram (EEG), nasal respiratory (RESP), arterial blood pressure (BP), and electrocardiogram (ECG) signals. Artifactual outliers are simulated across all four signal morphologies with one signal being “disrupted” at a time to allow for the study of differences in the effect of outliers based on the signal containing them. Multiple experiments are conducted with varying percentages of outlier samples. The values of features extracted from network segments containing artifactual outliers are compared with the respective values of features extracted from the original network segments to quantify the disruptive capacity of outliers.

Finally, the logistic regression classifier is tested in two configurations—a univariate, and a network-based multivariate configuration. The two configurations are deployed to allow for comparisons between the two approaches and identify benefits, as well as potential challenges when moving from univariate to multivariate analysis for the classification of network segments.

## 2. Methods

### 2.1. Stages of the Study

The research presented in this manuscript is conducted in the following stages:

- Network Formulation and Feature Extraction: The available physiological signal recordings are segmented in multivariate network segments. For each normal network segment, 16 different variants of artifactual network segments are produced, one for each experimental setup of interest, as described in Section 2.6. From each segment, a total of 15 univariate and multivariate features are extracted.
- Statistical Analysis: Conducted separately in each experimental setup, statistical analysis is applied to each extracted feature at the level of: separate feature distributions, pairs of feature distributions, and individual feature values, as described in Section 2.7.
- Artifactual Network Segment Detection: A univariate and multivariate network-based logistic regression classifier is trained and tested in each experimental setup to assess the capacity of both configurations in detecting artifactual network segments when outliers are present in different physiological signals and at varying percentages of occurrence.

**Figure 1:**
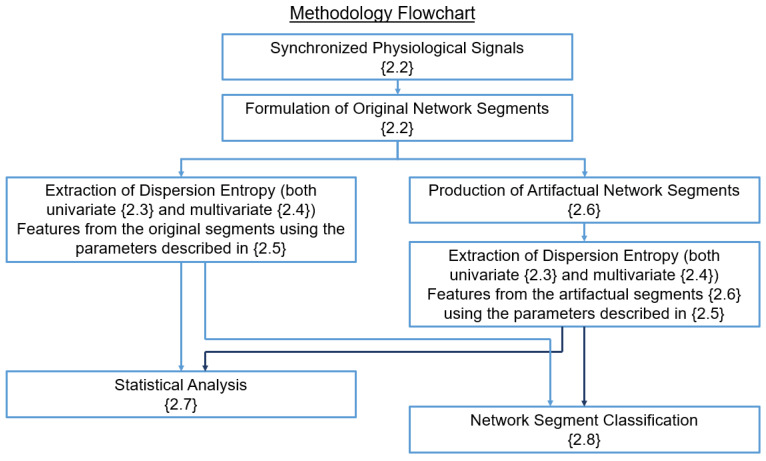
The methodological steps of the study are presented, with the sections corresponding to each step indicated in each block within {}. The arrows between each block indicate the outputs of a step that are used as inputs by the next one.

Figure 1 displays a flowchart where the methodological steps of the study are shown. The interconnections between steps indicate the way in which the outputs of one step are utilized as inputs by the following. The methodological steps are discussed in detail in the following sections.

### 2.2. Experimental Data and Preprocessing

For the formulation of a network based on multiple synchronised physiological recordings, the publicly available MIT-BIH Polysomnographic Database is chosen, which contains a total of 18 records of multiple physiological signals initially recorded for the evaluation of chronic obstructive sleep apnea syndrome and digitized at a sampling interval of 250 Hz [34,35]. For the purposes of this study, 11 records are selected based on the availability of complete and synchronised recordings of EEG, RESP, BP, and ECG signals.

Each signal recording is segmented in non-overlapping windows of 7500 samples, resulting in a total of 1463 physiological signal segments, 133 per record. Within the framework of network physiology, the extracted segments are analyzed in sets of 1463 multivariate “network segments” consisting of four synchronised windows, one from each physiological signal, following the process described in Section 2.5. The length of the window is chosen, after consulting the respective literature [11,12] to ensure that it is long enough as to allow a sufficient amount of samples for the effective calculation of the output DisEn values, while at the same time being short enough to provide an adequate temporal resolution for effective monitoring of the system. No further preprocessing is applied to the signals prior to the application of a mapping function and the respective DisEn algorithms.

### 2.3. Univariate Dispersion Entropy

DisEn arises from the integration of Shannon entropy with symbolic dynamics, aiming to quantify the degree of irregularity in an input signal segment, in low computational time, while achieving increased discrimination capacity [10,11]. Prior to the application of DisEn, an optional but recommended preprocessing step is the application of a non-linear mapping function to the input time-series. The process followed by the algorithm for the analysis of either the original or the mapped input univariate time-series $x_{j}\left(j=1,2,\ldots ,N\right)$ of length *N* is the following:

1. Production of a “quantised” time-series: A number of classes (*c*) are distributed along the amplitude range of the time-series, and each sample is allocated to the nearest respective class based on its amplitude. This results in the production of a "quantised" time-series $u_{j}\left(j=1,2,\cdots ,N\right)$.
2. Formulation of embedded vectors: An embedding dimension (*m*) and a time delay (*d*) are set for the creation of embedded vectors, $u_{i}^{m,c}=\left\{u_{i}^{c},u_{i+d}^{c},\cdots ,u_{i+(m-1)d}^{c}\right\}$, of length *m*, for each i=1,2,…,N−(m−1)d.
3. Mapping to dispersion patterns: Each embedded vector $u_{i}^{m,c}$ is mapped to a respective dispersion pattern $\pi_{v_{0}\cdots v_{m-1}}$ based on its corresponding classified samples. The number of potential unique dispersion patterns is $c^{m}$, as defined by the number of classes and the embedding dimension.
4. Calculation of Dispersion Pattern Relative Frequency: For each of the $c^{m}$ unique dispersion patterns, their relative frequency is calculated as follows:
  (1) $$\begin{array}{c} p\left(\pi_{v_{0}\cdots v_{m-1}}\right)=\frac{\#\{i\left|i\leq N-\left(m-1\right)d,u_{i}^{m,c}\;\mathrm{has}\;\mathrm{type}\;\pi_{v_{0}\cdots v_{m-1}}\right.\}}{\left(N-(m-1)d\right)}. \end{array}$$
5. Calculation of Univariate Dispersion Entropy: Utilizing the obtained relative frequencies, the time-series’ output DisEn value is calculated using the following equation [10,11], based on Shannon’s definition of entropy:
  (2) $$\begin{array}{c} DisEn\left(X,m,c,d\right)=-\sum_{\pi =1}^{c^{m}}p(\pi_{v_{0}\cdots v_{m-1}})\cdot \ln\left(p(\pi_{v_{0}\cdots v_{m-1}})\right) \end{array}.$$

Following the aforementioned steps, an input signal described by a single dispersion pattern would result in a minimum output DisEn value (i.e., 0) as opposed to one requiring the utilization of all possible dispersion patterns in equal probability, which would result in a maximum output value. An in-depth analysis concerning suggested mapping functions and optimisation of parameter values is available in [11].

### 2.4. Multivariate Dispersion Entropy

Multivariate Dispersion Entropy (mvDE) allows the multivariate quantification of DisEn from multiple input time-series. Similarly to its univariate equivalent, the preprocessing of each individual time-series using a mapping function is recommended. Assuming a multivariate set of *p*-input time-series $X={\left\{x_{k,i}\right\}}_{k=1,2,\cdots ,p}^{i=1,2,\cdots ,N}$ of length *N* each, the computational steps of mvDE are the following:

1. Production of univariate “quantised” time-series: In a process similar to its univariate variation, a number of classes (*c*) are distributed along the amplitude range of each time-series separately. For every time-series, their samples are allocated to their nearest respective class based on their amplitude. As a result, a quantised time-series $u_{j}\left(j=1,2,\cdots ,N\right)$ is produced for each respective input time-series, resulting in a set of *p*-quantised time-series $U={\left\{u_{k,i}\right\}}_{k=1,2,\cdots ,p}^{i=1,2,\cdots ,N}$.
2. Formulation of multivariate embedded vectors: For the production of multivariate embedded vectors, an embedding dimension *m* and a time delay *d* are set for construction of initially univariate embedded vectors of length *m* from each separate signal, similarly to the respective process for univariate DisEn. The univariate embedded vectors are then combined in sets of *p*-synchronised vectors, one from each input signal. The vectors within each group are then serially concatenated for the production of respective multivariate embedded vectors Z(j), of length mp, for each j=1,2,…,N−(m−1)d.
3. Mapping to multiple dispersion patterns: In mvDE, each embedded vector is mapped to multiple dispersion patterns. Each subset of *m* elements in Z(j) is accessed, following all possible mpm combinations. This formulates ϕq(j)(q=1,…mpm) subvectors, that are then mapped to their corresponding $\pi_{v_{0}\ldots v_{m-1}}$ dispersion pattern. As a result, the total number of dispersion pattern instances is (N−(m−1)d)mpm and the number of unique dispersion patterns is $c^{m}$.
4. Calculation of Dispersion Pattern Relative Frequency: The relative frequency of each dispersion pattern is calculated in a manner similar to Equation (1), but with the correct adjustment for the increased number of instances:
  (3) p(πv0…vm−1)=#{jj≤N−(m−1)d,ϕq(j)hastypeπv0…vm−1}(N−(m−1)d)mpm.
5. Calculation of Multivariate Dispersion Entropy: The extracted relative frequencies are used for the calculation of the respective multivariate DisEn value based on Shannon’s definition of entropy.

(4) $$\begin{array}{c} mvDE\left(X,m,c,d\right)=-\sum_{\pi =1}^{c^{m}}p(\pi_{v_{0}\ldots v_{m-1}})\cdot \ln\left(p(\pi_{v_{0}\ldots v_{m-1}})\right) \end{array}.$$

The algorithmic variation utilised in this study and described in the aforementioned steps is the fourth and, by its designers, recommended variation of the mvDE algorithm. Further details concerning the operation of the algorithm, its other variations, and the comparative evaluation of their performance are available in [12].

### 2.5. Extraction of DisEn Features

For each normal network segment, a total of 15 features are extracted, four of which are univariate, one for each physiological signal, and 11 of which are multivariate, based on the subnetwork combinations of: EEG-RESP, EEG-BP, EEG-ECG, RESP-BP, RESP-ECG, BP-ECG, EEG-BP-ECG, RESP-BP-ECG, EEG-RESP-BP, EEG-RESP-ECG, and EEG-RESP-ECG-BP.

In the case of univariate features, for each signal, its respective 7500 sample window is fed as input to the univariate DisEn algorithm. For each multivariate feature, the respective combination of synchronised 7500 sample windows is fed as input to the multivariate DisEn algorithm for its calculation.

Table 1 displays the parameter values which are the same for univariate and multivariate DisEn. The values were selected after consulting the performance benchmarks provided in the respective studies [11,12] and taking into consideration the number of 7500 samples contained within each input window. As a preprocessing step, each individual time-series is mapped using the normal cumulative distribution function (NCDF).

### 2.6. Production of Artifactual Network Segments

Within the scope of this study, artifactual outlier samples are simulated across all four signal morphologies—EEG, RESP, BP, and ECG—with one signal being “disrupted” at a time. Furthermore, the percentage of samples being outliers is determined by the percentage factor *P* whose value varies across experimental setups in the levels of 0.1%, 0.5%, 1%, and 5%. As a result, a total of 16 experimental setups are formulated, containing the 1463 normal network segments and a corresponding variation of 1463 artifactual network segments.

**Figure A1:**
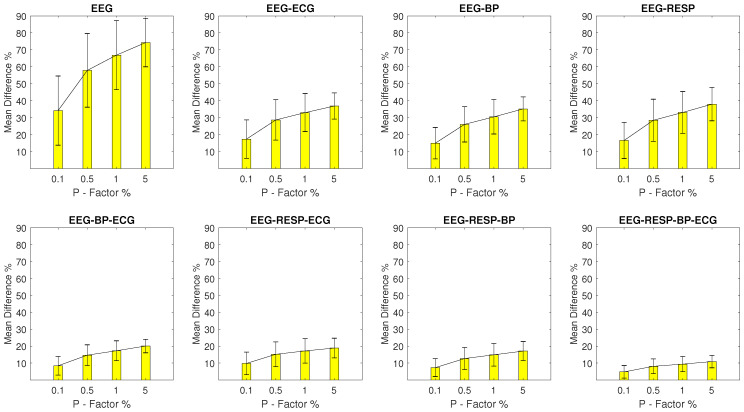
The μ and σ of the percentage difference are shown for each artifactual feature distribution across experimental setups where the EEG signal of the network contains a percentage of outliers determined by the corresponding *P*-Factor. These results correspond to experimental setups with outliermean=±4×max|amplitude|.

**Figure A2:**
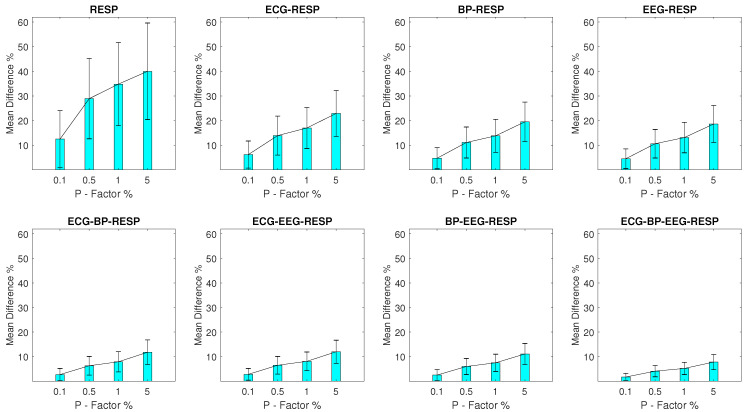
The μ and σ of the percentage difference are shown for each artifactual feature distribution across experimental setups where the RESP signal of the network contains a percentage of outliers determined by the corresponding *P*-Factor. These results correspond to experimental setups with outliermean=±4×max|amplitude|.

**Figure A3:**
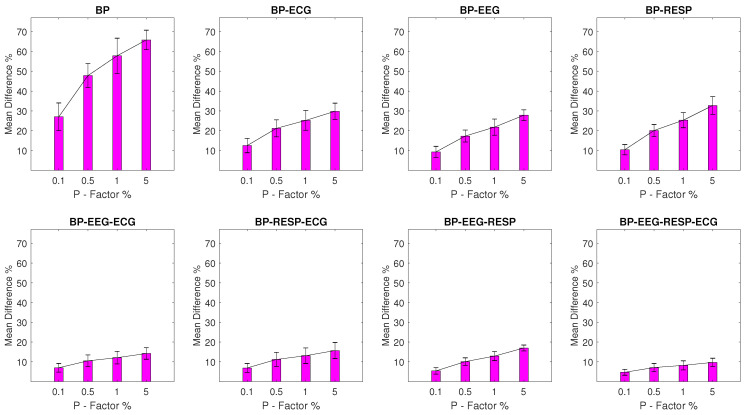
The μ and σ of the percentage difference are shown for each artifactual feature distribution across experimental setups where the BP signal of the network contains a percentage of outliers determined by the corresponding *P*-Factor. These results correspond to experimental setups with outliermean=±4×max|amplitude|.

**Figure A4:**
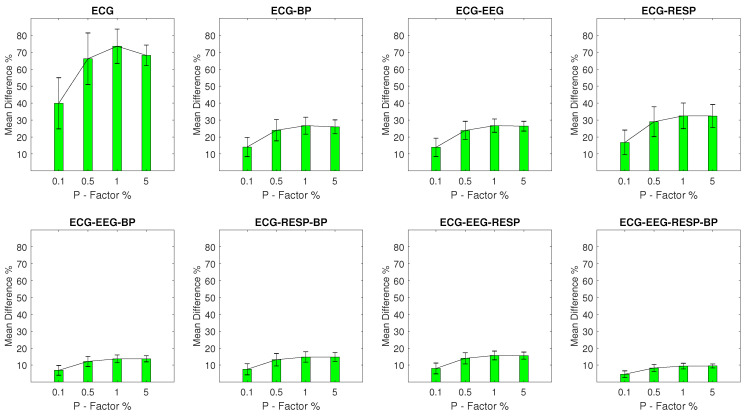
The μ and σ of the percentage difference are shown for each artifactual feature distribution across experimental setups where the ECG signal of the network contains a percentage of outliers determined by the corresponding *P*-Factor. These results correspond to experimental setups with outliermean=±4×max|amplitude|.

The process through which the 1463 artifactual network segments of each experimental setup are produced, is the following:

1. Marking of Outlier Samples: Based on the percentage factor *P*, a percentage of samples are uniformly drawn from each 7500-sample window, and their amplitude is replaced with a value in the outlier amplitude range.
2. Setting the Value of Outlier Samples: The amplitude of each outlier is obtained from a Gaussian distribution with a standard deviation (σ) equal to the absolute maximum amplitude of each signal: σ=max|amplitude|. Concerning the distribution mean (μ), there are two choices to be considered. The first choice, which is in alignment with the simulation processes followed by previous studies testing the effect of outlier samples in the performance of ApEn, SampEn, and univariate DisEn [23,24,25], would be to ensure that outlier values are outside the physiological range of each recorded signal while maintaining the amplitude boundaries of respective sensing equipment, leading to a distribution μ equal to outliermean=±4×max|amplitude|. The second choice would be to set a lower distribution μ to ensure that a minority of outliers remain within physiological range to also cover certain scenarios where sensor miscalibration and recording interferences could produce outliers of the respective magnitude, and for that purpose, a μ equal to outliermean=±2×max|amplitude| would be set. In order to provide results that are comparable to previous studies while at the same time covering all possible scenarios, each experimental setup is replicated for both outlier μ; however, since the second case covers a wider range of scenarios, its corresponding results are reported and discussed in detail, while the results of the first case (outliermean=±4×max|amplitude|) are available in the Appendix, in Figure A1, Figure A2, Figure A3 and Figure A4 and Table A1, Table A2, Table A3 and Table A4 of this manuscript. In all experimental setups, the sign of half the outliers is set to positive and the other half to negative, following random assignment.
3. Calculation of Artifactual DisEn Features: For each experimental setup, the corresponding 1463 artifactual network segments are used to calculate the respective univariate and multivariate artifactual DisEn Features based on the same process that was followed for the normal network segments in Section 2.5.

To facilitate the reproducibility of the presented study, the function used for the simulation of outlier samples is made publicly available as Supplementary Material.

### 2.7. Statistical Analysis

To quantify the disruptive capacity of outlier samples in the accuracy of extracted network features, the following three-stage statistical analysis is applied.

1. Kolmogorov–Smirnov Test: Initially, each feature distribution is standardised and compared to a standard normal distribution using the Kolmogorov–Smirnov Test.
2. Mann–Whitney *U* Test: At the second stage, and after consulting the results of the Kolmogorov–Smirnov test, the Mann–Whitney *U* test is chosen to compare each feature distribution extracted from artifactual network segments with its corresponding feature distribution extracted from the respective normal network segments, to verify statistically significant differences between the distributions of each pair.
3. Mean Percentage Difference: Finally, for each DisEn feature extracted from an artifactual network segment, the absolute percentage difference from its original value, the one calculated from the respective network segment without outliers, is calculated. To provide a summary for every feature extracted during each experimental setup, its mean percentage difference (MPD) and σ of the percentage difference are calculated.

### 2.8. Artifactual Network Segment Detection

For the detection of artifactual network segments, a logistic regression classifier is applied in two configurations, a univariate and a multivariate one. The univariate configuration is utilising, from each network segment, only the the four DisEn features that are extracted using the physiological signal segments as separate input to the univariate DisEn algorithm, while the multivariate configuration utilises all the available 15 features of each network segment. The choice to implement two separate algorithmic configurations is made with the following aims in mind:

- To derive insights concerning the potential benefits but also challenges that arise when moving from univariate to multivariate analysis for network segment classification.
- To identify differences in classification performance, for both configurations, based on the physiological signal containing outlier samples in each experimental setup.

For this purpose, both algorithmic configurations were tested under the same 16 experimental setups. Each setup contains features extracted from a total of 2926 segments, out of which 1463 are the original network segments, and the other 1463 are their artifactual variations, as determined by the parameters of the experiment. As mentioned in Section 2.2, the 1463 network segments correspond to 11 records, or 133 segments per record. The segments selected for training and testing during each experimental setup are selected in the following two data splits.

The first data split is done at the record level, with the first nine records used for training and the last two for testing purposes. This is done to ensure that the training of the classifier is done on different patients than the ones it is tested on, and therefore, its recorded performance is patient-agnostic. This leads to the feature sets of a total of 2394 segments (1197 normal vs. 1197 artifactual ones) being used for training, and a total of 532 feature sets (266 normal vs. 266 artifactual ones) being used for testing.

At this point, a second data split is introduced in the training set. It is important to consider that in a field application, the classifier would never have access to the exact same network segments in both normal and artifactual variations. For this reason, only half, or 1197 training sets are used, the first 599 of which correspond to feature sets of a normal network segment, while the other 598 correspond to different artifactual ones. As a result, for each experimental setup, both classifier configurations are trained on 1197 distinct training feature sets and tested on 532 testing feature sets.

Finally, the performance of each configuration for a certain experimental setup is calculated as the percentage of correct network segment classifications observed for each configuration when applied to the respective testing dataset.

## 3. Results

### 3.1. Kolmogorov–Smirnov and Mann–Whitney U Test Results

With 16 experimental setups and 15 features extracted from the network segments of each setup, a total of 240 feature distributions are produced corresponding to artifactual network segments, alongside 15 feature distributions corresponding to normal network segments. For the outliers with a μ of: outliermean=±2×max|amplitude|, 211 out of the total 255 distributions displayed a statistically significant difference from a normal distribution after being standarised, rejecting the null hypothesis with *p*-value < 0.05. For outliers with a μ of outliermean=±4×max|amplitude|, 212 out of the 255 distributions rejected the null hypothesis at *p*-value < 0.05. Taking into consideration the non-Gaussian nature of most feature distributions, the Mann–Whitney *U* test is selected to compare in pairs, the feature distributions extracted from artifactual network segments with the feature distributions extracted from the corresponding normal network segments.

Since in each experimental setup, only one of the physiological signals contains outliers, out of the 15 total features extracted per network segment, only eight of these features were extracted from signal combinations that include the “artifactual” signal. As a result, it is expected that in each experimental setup these eight feature distributions will display a statistically significant difference when compared to the distributions of features extracted from the respective normal segments. No significant difference is expected for the seven feature distributions that do not include the "artifactual" signal.

As expected, for both categories of outlier distributions with different μ magnitude, and for all experimental setups, all feature distributions extracted from a combination of segments containing outliers have a statistically significant difference to the original feature distributions, rejecting the null hypothesis with *p* values $<10^{-12}$, while no statistical difference is observed in the rest of the pairs.

### 3.2. Disruption of Feature Values Across Experimental Setups

The experimental setups presented in this study contain networks within which one physiological signal contains artifactual outliers. As a result, while network features that are not extracted from network segments containing outliers remain unaffected, the values of rest of the features display significant MPD, as highlighted by the following results. The Section 3.2.1, Section 3.2.2, Section 3.2.3 and Section 3.2.4 present in detail the results for outliers with outliermean=±2×max|amplitude|. Furthermore, concerning outliers with outliermean=±4×max|amplitude|, the corresponding results are available in the appendix, in Figure A1, Figure A2, Figure A3 and Figure A4, and tend to follow similar patterns to the outliers of the first category, but with constant increase in the overall values of MPD, which is expected considering the increased deviation of the mean outlier amplitude from the physiological amplitude range.

#### 3.2.1. Setups with EEG Outliers

**Figure 2:**
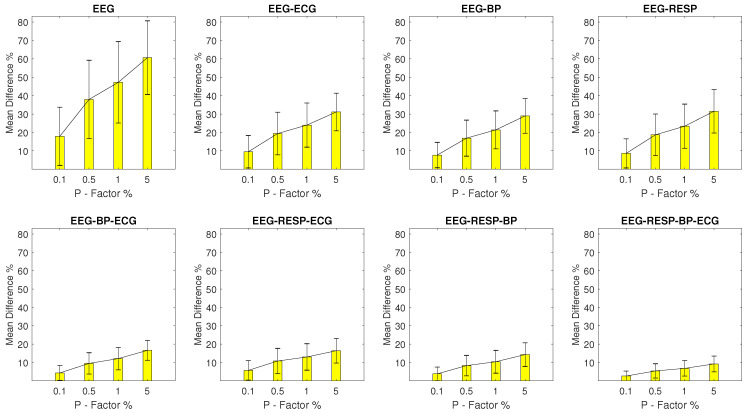
The μ and σ of the percentage difference are shown for each artifactual feature distribution across experimental setups where the EEG signal of the network contains a percentage of outliers determined by the corresponding *P*-factor.

The MPD values for setups with EEG outliers are shown in Figure 2. For the univariate feature, the MPD ranges from a minimum value of 17.9% with a σ of 15.8% observed at a *P* factor of 0.1%, to a maximum value of 60.6% with a σ of 20% observed at a *P* factor of 5%. Bivariate features display a maximum MPD of 31.5% and a σ of 11.8% observed for the feature extracted from synchronised segments of the EEG and RESP signals at a *P* factor of 5%, while for the rest of the features, the MPD values do not surpass 16.6%. It is important to note the continuous decrease of MPD observed as the number of signals forming the network segments from which a feature is extracted, increases.

#### 3.2.2. Setups with RESP Outliers

**Figure 3:**
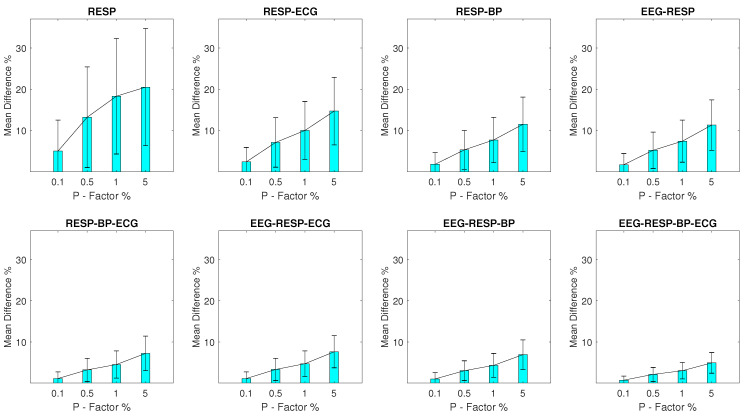
The μ and σ of the percentage difference are shown for each artifactual feature distribution across experimental setups where the RESP signal of the network contains a percentage of outliers determined by the corresponding *P*-factor.

For setups with RESP outliers, a noteworthy change is observed in the results shown in Figure 3. Significant but substantially smaller MPD values are observed for the univariate feature when compared to the EEG outlier setups, with a minimum MPD of 5% and a σ of 7.5% observed for a *P* factor 0.1% and a maximum MPD of 20.5% and a σ of 14.2% observed for a *P* factor of 5%. The bivariate features follow closely with a maximum MPD of 14.7% and a σ of 8.2% observed for the feature extracted from synchronised segments of the RESP and ECG signals, at a *P* factor of 5%, while the MPD values for the rest of the features do not surpass 7.6%.

#### 3.2.3. Setups with BP Outliers

**Figure 4:**
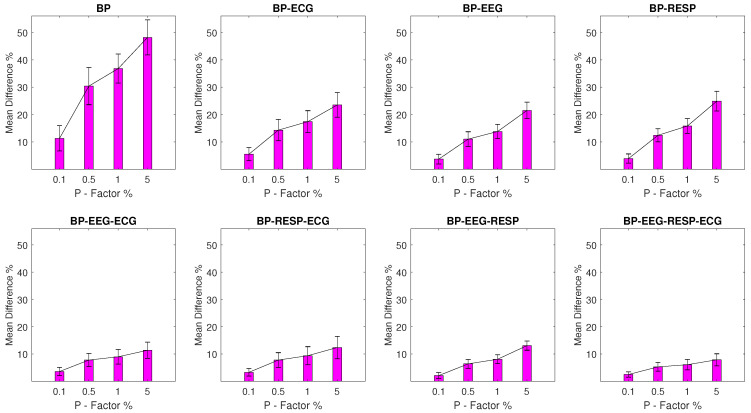
The μ and σ of the percentage difference are shown for each artifactual feature distribution across experimental setups, where the BP signal of the network contains a percentage of outliers determined by the corresponding *P*-factor.

The MPD values for experimental setups with BP outliers are shown in Figure 4 and seem to follow a similar pattern to the one observed for the EEG and RESP setups. The BP univariate feature displays significant value disruption with a minimum MPD of 11.3% and a σ of 4.6% for a *P* factor of 0.1% increasing to a MPD of 48.2% with a σ of 6.4% for a *P* factor of 5%. For bivariate features, a maximum MPD of 24.9% and a σ of 3.6% are observed for the feature extracted from synchronised segments of the BP and RESP signals at a *P* factor of 5%, while the rest of the features follow with MPD values that do not exceed 13.1%.

#### 3.2.4. Setups with ECG Outliers

**Figure 5:**
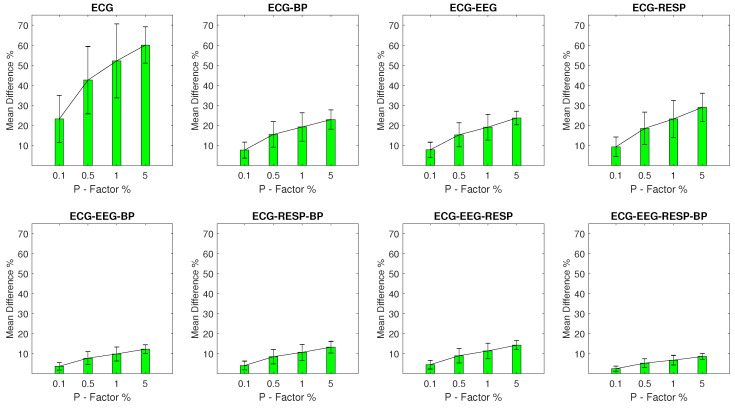
The μ and σ of the percentage difference are shown for each artifactual feature distribution across experimental setups where the ECG signal of the network contains a percentage of outliers determined by the corresponding *P*-factor.

Finally, the MPD values for experimental setups with outlier samples contained in the ECG signal are shown in Figure 5 following the same pattern. The univariate feature extracted from the ECG signal contains the highest MPD range with a minimum value of 23.2% and a σ of 11.7% for a *P* factor of 0.1%, increasing to 60.1% with a σ of 9.1% for a *P* factor of 5%. Bivariate features follow with a significant reduction in disruption. Their maximum MPD is observed in the case of the feature extracted from synchronised segments of the ECG and RESP signals with value of 29% and a σ of 7.1% observed at a *P* factor of 5%. The rest of the features follow with MPD values that are lower than 14.2% across all respective setups.

### 3.3. Network Segment Classification Results

As indicated by the MPD results, outliers have a significant effect in the values of the extracted features, with the univariate feature having the largest deviations from the original values. It would therefore be important to verify whether these deviations can be used to detect artifactual network segments, in the case of the univariate classifier, and whether the inclusion of multivariate features improves performance or introduces disruptive noise. The performances of both the univariate and multivariate classifiers are reported for the respective experimental setups which are grouped together based on the signal of the network containing outliers, following the same format as Section 3.2. In the following Section 3.3.1, Section 3.3.2, Section 3.3.3 and Section 3.3.4, the results for outliers with outliermean=±2×max|amplitude| are presented in detail, while the results for the outliers with outliermean=±4×max|amplitude|, are available in the appendix, Table A1, Table A2, Table A3 and Table A4.

#### 3.3.1. Classification Performance with EEG Outliers

In the case of EEG outliers, a pattern of performance improvement is observed when moving from univariate to multivariate classification, as shown in Table 2. Initially, the multivariate classifier significantly outperforms the univariate one with correct classification percentages of 88.7% compared to the univariate 70.3% for a *P* factor 0.1% and of 97.2% compared to 88.5% for a *P* factor of 0.5%. Eventually, the univariate and multivariate classifiers reach equivalent performance levels for *P* factors of 1% and 5%.

#### 3.3.2. Classification Performance with RESP Outliers

For experimental setups with RESP outliers, limited effectiveness is initially observed for both classifiers, while effective performance is achieved when moving from the univariate to the multivariate model for a *P* factor of 5%. As shown in Table 3, both classifiers display performance that does not surpass a percentage of correct classifications of 56% for a *P* factor of 0.1%. However, for a *P* factor of 5%, the multivariate model achieves a percentage of 96.2% of correct classifications, significantly outperforming the univariate one with a respective percentage of 76.5%.

#### 3.3.3. Classification Performance with BP Outliers

For experimental setups with BP outliers, both univariate and multivariate classifiers achieve similar and effective performance as displayed in Table 4. The correct classification percentages are in the range of 94% to 100% for the univariate classifier and above 99% for the multivariate one. In this case, artifactual segments are detected even in significantly low percentages of outliers, indicating that the signal in which the outliers are located plays an important role in the correct classification of the corresponding network segments.

#### 3.3.4. Classification Performance with ECG Outliers

Finally, in the case of ECG outliers, a different pattern is observed when moving from a univariate to a multivariate classifier. As shown in Table 5, for a *P* factor of 0.1%, a performance boost is noted from 61.8% of correct classifications for the univariate classifier, increasing to 68.6% for the multivariate one. However, for larger *P* factors, the univariate classifier is constantly outperforming the multivariate one with substantial effectiveness, considering the correct classification percentage range of 94.9% to 100% as opposed to the multivariate performance range of 74.4% to 95.7%. This indicates that after a certain threshold of outliers in the network, the univariate classifier is effective, while the addition of multivariate features adds noise that substantially reduces the corresponding performance of the multivariate one.

## 4. Discussion

As part of this study, we quantified the disruptive capacity of signal-specific outliers in the values of all possible univariate and multivariate DisEn features extracted from corresponding network segments. Each network segment consists of four synchronised physiological signal segments: EEG, RESP, BP, and ECG, resulting in a total of 16 experimental setups, with each setup being defined by the signal containing the artifactual outliers and the percentage of samples set as outliers as specified by the corresponding *P* factor, with possible values being: 0.1%, 0.5%, 1%, and 5%. Furthermore, for all 16 experimental setups, a univariate and a multivariate logistic regression classifier is trained and tested for the detection of artifactual network segments, with the percentage of correct segment classifications being reported for each setup (in Table 2, Table 3, Table 4 and Table 5).

### 4.1. Robustness of Multivariate Network Features to Univariate Outliers

Based on the results presented in Section 3.2, a pattern can be observed in the recorded MPD values. The multivariate features have significantly lower values of MPD from the correct feature values, when compared to the corresponding MPD of the univariate feature for each experimental setup. This was particularly noticeable for setups with high *P* factor values, such as 1% or 5%, when outliers are present in the EEG and ECG signals, with the univariate MPD surpassing the corresponding multivariate MPD by at least 23%. Furthermore, the MPD becomes lower as the number of signals forming the network segments from which a feature is extracted, increases. The feature extracted from all four available signals in a network segment has consistently the lowest MPD values across all experimental setups. Finally, in the case of experimental setups with outliers in the RESP signal, while the same pattern persists, the reduction of the value of the MPD when moving from univariate to multivariate features is significantly smaller. This further indicates that signal-specific characteristics affect the disruptive capacity of outliers, a subject that is discussed in further detail in Section 4.2.

When comparing the operation of univariate and multivariate DisEn, a core difference that provides a relative robustness to multivariate features when compared to univariate ones concerns the number of quantised samples formulating the dispersion patterns from which the value of the corresponding feature is calculated. For the univariate DisEn algorithm, as indicated in Section 2.3, the embedding dimension (*m*) defines the number of quantised samples used to create dispersion patterns [10,11]. In this study, m=3 for both the univariate and multivariate algorithms. However, as mentioned in Section 2.3, in the case of multivariate features, the number of quantised samples formulating a dispersion pattern increases with the number of signals used for its extraction. As a result, the effect of outlier samples in the formulation of the corresponding dispersion patterns is reduced, leading to a reduction in their capacity to disrupt the calculation of the respective DisEn value.

Therefore, while the outliers still led to a significant disruption in the distribution of values for the corresponding multivariate features, as indicated by the results of the Mann–Whitney *U* test reported in Section 3.1, the relative robustness of multivariate features when compared to univariate ones indicates a potential advantage of multivariate network-based methodologies for respective applications. It would be worthwhile to expand on this study with the replication of equivalent experiments utilising other entropy quantification algorithms, such as the univariate and multivariate variations of PEn [13,14] and SampEn [16,17] to verify whether the robustness of multivariate features to univariate outliers is persistent across different entropy quantification methodologies.

### 4.2. Disruptive Effect of Outliers Across Physiological Signals

As indicated by the results presented in Section 3.2 and mentioned in Section 4.1, significant changes in the effect of outliers on the value of the extracted univariate features are observed, based on the signal containing the corresponding outliers. The disruption is more significant when outliers are present in the EEG and ECG signals, with slightly smaller disruption observed when outliers are present in the BP signal and significantly smaller for outliers in the RESP signal. A similar pattern was observed in a previous study, focusing on the development of univariate DisEn variations that would be robust to outliers [25]. When outliers are present in a signal segment, they tend to disrupt the process of allocating classes across the amplitude range of the segment [10,12] by significantly expanding it based on the outliers’ amplitudes. As a result, the amount of classes allocated in the physiological range is significantly reduced, leading to a reduction of dispersion patterns representing physiological dynamics in the signal, and therefore an overall reduction of the calculated DisEn values. Signals such as the ECG [36] and EEG [37] contain higher-frequency components leading to rapid fluctuations in the amplitude of each signal, relative to the BP [38] and especially the RESP [39,40] signals. As a result, the observed decrease in DisEn values differs from signal to signal, resulting in the observed difference in MPD magnitudes.

It is important to notice that the same pattern is not observed to the same degree in the case of multivariate features. As discussed in Section 4.1, the multivariate features display a certain robustness to the effect of outliers based on the number of signals formulating the network segments from which they are extracted. No major deviations, in the values of MPD, are observed among the same multivariate features across different experimental setups where the signal containing the outliers, changes. These findings indicate that the potential combination of univariate DisEn algorithms modified to be robust to the effect of outliers [25] with the current multivariate DisEn variation [12], could provide an effective entropy quantification interface for the extraction of features from network segments.

### 4.3. Performance Comparison in Artifactual Segment Detection

The classification performance observed across experimental setups and presented in Section 3.3.1, Section 3.3.2, Section 3.3.3 and Section 3.3.4, indicates promising results for the detection of artifactual network segments. However, strong differences that should be taken into consideration are observed in the performance of the univariate and multivariate classifiers based on the signal containing the artifactual outliers. In the case of EEG and RESP experimental setups, the multivariate classifier outperformed the univariate one, while significant performance differences were observed between the two groups of experimental setups. In the case of the EEG signal, significant performance was achieved by the multivariate classifier even for low *P*-factor values, such as 0.5%, while the univariate classifier reached similar levels of performance for *P*-factor values of 1% and 5%. However, the RESP outliers proved much more challenging in the detection of the corresponding network segments, with only the multivariate classifier achieving effective performance at a *P* factor of 5%. It is important to consider that the reduced performance in the case of the experimental setups with RESP outliers was expected, especially in the case of the univariate classifier, when considering that the corresponding outliers had significantly lower disruptive capacity when compared to experimental setups with outliers in other signals, as quantified in Section 3.3.2. In the case of BP outliers, no significant performance differences were noted between the univariate and multivariate models with both classifiers displaying effective performance, as mentioned in Section 3.3.3.

However, the case of ECG outliers highlights an important challenge when deploying architectures that utilise multiple multivariate features. For *P* factor values of 0.5% and higher, the univariate model is not just highly effective at classifying network segments, but also outperforms the multivariate one, indicating that in this case, the multivariate features add noise that reduces the performance of the multivariate model. This highlights the necessity of utilising a machine-learning architecture that would be robust to potential noise added by the utilisation of multiple features, through effective feature selection [41,42,43]. This is particularly important for future applications of network physiology aiming not just at the assessment of data quality, but also at the extraction of physiological insights from networks [44,45,46], since in those types of applications, the most informative features will be harder to detect due to the dynamics of interest having the potential to occur at any level of multivariate interaction, as opposed to starting from outliers which initially occur at one of the univariate signals during recording.

### 4.4. Limitations of Current Study and Future Work

Throughout the experimental setups of this study, it is important to note that, as mentioned in Section 2.6, only one signal at a time contained artifactual outliers. This design choice for the experiments of the study was made in order to prioritise experimental setups that would provide insights, not just about the effect of artifactual outliers in the quantification processes of DisEn, but also about how these effects differ based on which physiological signal of the network contains the outliers. Consequently, this study could be further expanded through the conduction of experiments where outliers are present in more than one signal of the network at a time, this is expected to lead to higher MPD observed for multivariate features, and therefore, even better classification performance in the detection of artifactual network segments when utilising a multivariate classifier.

Furthermore, while the simulated artifactual outliers presented in this study were in alignment with previous research in the field [24,25], it would be important to replicate this study and especially the classification of artifactual network segments, utilising data sets with annotated real-world artifactual segments to further assess the applicability of the method as a deployed data quality control tool.

Finally, as indicated by the results presented in Section 3.3 and discussed in Section 4.3, the logistic regression classifier was not able to appropriately utilise the multivariate features in the case of experimental setups with ECG outliers, leading to reduced performance when compared to the univariate one. Therefore, both for the purpose of artifactual segment detection and for future applications of network physiology, it would be important to expand on this study by designing and implementing classification architectures capable of effective feature selection.

## 5. Conclusions

This study investigated and quantified the effect of artifactual outlier samples in the accuracy of univariate and multivariate DisEn features extracted from network segments consisting of four synchronised physiological signals. Furthermore, it presented a proof-of-concept artifactual segment detection tool deployed in univariate and multivariate configurations using the corresponding extracted features.

The results indicate that the distribution of values for each feature extracted from a network segment containing artifactual outliers is significantly altered. The largest magnitude of disruption is observed in univariate features with an MDP value in the range of 20–48% for most experimental setups, while the multivariate features display a relative robustness, which increases based on the number of signals from which they are extracted. The feature extracted from all four available signals in a network segment displays an MDP value that remains lower than 10% across experimental setups. The classification results of the study indicate that the univariate classifier performance surpasses 90% of correct segment classifications for the majority of experimental setups. A strong exception are the setups with outliers in the RESP signal where a performance of 90% correct segment classifications is surpassed only with the multivariate classifier for a *P* factor of 5%. The multivariate classifier outperforms the univariate one in setups with EEG and RESP outliers, but underperforms when compared to the univariate one in the case of ECG outliers. These results highlight the importance of using a machine learning architecture capable of effective feature selection when moving from univariate to multivariate analysis within the framework of network physiology.

Finally, the changes observed both in terms of the percentage differences and the classification effectiveness when comparing across experimental setups in which outliers are present in different signals indicate that, in alignment with prior research [25], the characteristics of each physiological signal should be taken into consideration when assessing the impact of outlier samples in the process of entropy quantification.

## Figures and Tables

**Table 1:** Parameter values for univariate and multivariate DisEn.

Parameter	Symbol	Value
Embedding Dimension	*m*	3
Number of Classes	*c*	9
Time Delay	*d*	1

**Table 2:** Percentage of correct network segment classifications for univariate and multivariate classifiers when tested on experimental setups, with outliers located in the EEG signal of the network.

EEG	Univariate	Multivariate
0.1%	70.3%	88.7%
0.5%	88.5%	97.2%
1%	97.7%	98.5%
5%	99.1%	99.1%

**Table 3:** Percentage of correct network segment classifications for univariate and multivariate classifiers when tested on experimental setups with outliers located in the RESP signal of the network.

RESP	Univariate	Multivariate
0.1%	55.8%	55.6%
0.5%	64.1%	67.5%
1%	72.6%	71.2%
5%	76.5%	96.2%

**Table 4:** Percentage of correct network segment classifications for univariate and multivariate classifiers when tested on experimental setups with outliers located in the BP signal of the network.

BP	Univariate	Multivariate
0.1%	94%	99.1%
0.5%	100%	99.6%
1%	99.4%	100%
5%	100%	100%

**Table 5:** Percentage of correct network segment classifications for univariate and multivariate classifiers when tested on experimental setups with outliers located in the ECG signal of the network.

ECG	Univariate	Multivariate
0.1%	61.8%	68.6%
0.5%	94.9%	74.4
1%	97%	80.8
5%	100%	95.7

## Data Availability

The MIT-BIH Polysomnographic Database used for this study is publicly available on PhysioNet: https://doi.org/10.13026/C23K5S (accessed on 11 February 2021).

## Supplementary Materials

To facilitate the reproducibility of the presented study, the function used for the simulation of outlier samples is published in: http://doi.org/10.5281/zenodo.4530360 (accessed on 11 February 2021). The IDs of the records utilised from the publicly available MIT-BIH Polysomnographic Database, are the following: slp03, slp04, slp14, slp16, slp32, slp37, slp41, slp45, slp48, slp59, slp66. From each record, the samples with indexes in the range of 1 to 997500 were used.

## Abbreviations

The following abbreviations are used in this manuscript:

TDS	Time Delay Stability
DisEn	Dispersion Entropy
PEn	Permutation Entropy
ApEn	Approximate Entropy
SampEn	Sample Entropy
FuzzyEn	Fuzzy Entropy
EEG	Electroencephalogram
RESP	Nasal Respiratory
BP	Arterial Blood Pressure
ECG	Electrocardiogram
MPD	Mean Percentage Difference

## Appendix B

**Table A1:** Percentage of correct network segment classifications for univariate and multivariate classifier when tested on experimental setups with outliers located in the EEG signal of the network.

EEG	Univariate	Multivariate
0.1%	84.2%	96.6%
0.5%	98.9%	99.2%
1%	99.2%	98.9%
5%	99.6%	99.6%

**Table A2:** Percentage of correct network segment classifications for univariate and multivariate classifier when tested on experimental setups with outliers located in the RESP signal of the network.

RESP	Univariate	Multivariate
0.1%	63.7%	67.1%
0.5%	83.8%	76.3%
1%	89.1%	78.8%
5%	90.8%	95.1%

**Table A3:** Percentage of correct network segment classifications for univariate and multivariate classifier when tested on experimental setups with outliers located in the BP signal of the network.

BP	Univariate	Multivariate
0.1%	99.4%	99.4%
0.5%	100%	100%
1%	100%	100%
5%	100%	100%

**Table A4:** Percentage of correct network segment classifications for univariate and multivariate classifier when tested on experimental setups with outliers located in the ECG signal of the network.

ECG	Univariate	Multivariate
0.1%	95.5%	73.1%
0.5%	100%	91%
1%	100%	97.2%
5%	99.8%	100%
